# Connexin43 and AMPK Have Essential Role in Resistance to Oxidative Stress Induced Necrosis

**DOI:** 10.1155/2017/3962173

**Published:** 2017-11-27

**Authors:** Chunshan Zhao, Jinnv Fang, Chunguo Li, Min Zhang

**Affiliations:** ^1^Beihua University, Jilin, Jilin, China; ^2^Department of Preventive Medicine, College of Medicine, Yanbian University, Yanji, Jilin, China; ^3^Jilin City Central Hospital, Jilin, Jilin, China

## Abstract

Reactive oxygen species (ROS) induced oxidative stress leads to cell damage and neurological disorders in astrocytes. The gap junction protein connexin43 (Cx43) could form intercellular channels in astrocytes and the expression of Cx43 plays an important role in protecting the cells from damage. In the present study, we investigated the contribution of Cx43 to astrocytic necrosis induced by the ROS hydrogen peroxide (H_2_O_2_) and the mechanism by which AMPK was involved in this process. Fluorescence microscopy, flow cytometry, and western blot were used quantitatively and qualitatively to determine the cell apoptosis, necrosis, and protein expression. Lack of Cx43 expression or blockage of Cx43 channels resulted in increased H_2_O_2_-induced astrocytic necrosis, supporting a cell protective effect of functional Cx43 channels. Our data suggest that AMPK is important for Cx43-mediated ROS resistance. Inhibition of AMPK activation results in reduction of necrosis and ROS production. Taken together, our findings suggest that the role of Cx43 in response to H_2_O_2_ stress is dependent on the activation of AMPK signaling pathways and regulates ROS production and cell necrosis.

## 1. Introduction

Astrocytes are the most abundant nonneuronal cell type in the central nervous system [1]. They play an important role in glutamate uptake, potassium ion buffering, and antioxidant protection for neurons [2, 3]. The gap junction protein connexin43 (Cx43) is essential for the function of astrocytes and highly expressed inside the cell [4]. Cx43 can form gap junction channels as well as hemichannels. Gap junctions allow the passive intercellular diffusion of small molecules [5]. Oxidative stress is a result of the imbalance between reactive oxygen species (ROS) production and antioxidant activity. High level of ROS can also be generated under various conditions, leading to potential damage [6]. Cx43 expression in astrocytes behaved as the center of antioxidative stress [7]. Previous research indicated that Cx43 expression protected the C6 cells from ROS H_2_O_2_ stress [3]. What is more, knockdown of Cx43 increases cell death induced by H_2_O_2_. A signaling pathway which leads to caspase-3 activation is involved in this protection [8]. However, the mechanism underlying this Cx43-mediated ROS resistance to cell apoptosis and necrosis in astrocytes has not been well distinguished and quantified. The upstream activator of mechanistic target of rapamycin (mTOR) plays an important role in cell growth and apoptosis, mTOR activity is regulated by many kinases, and AMP-activated protein kinase (AMPK) is the most crucial one in this process. Phosphorylation of AMPK is likely to activate this signal pathway, and more existing regulators need to be discovered. Therefore, we investigated if H_2_O_2_-induced cell damage is owing to apoptosis or necrosis and AMPK activity in the process as well. In the present study, we used MTT assay and fluorescence microscopy to examine the role of Cx43 expression and activity in H_2_O_2_-induced astrocytic death. Further, the flow cytometry indicated that most of the cell damage was necrosis during the H_2_O_2_ stress, and it is well correlated with the expression of Cx43. Phosphorylation of AMPK is involved in H_2_O_2_ stress and ROS production. Specific inhibitor of AMPK could reduce the amount of cell caused by necrosis and decrease ROS production. Altogether, we concluded that Cx43 expression is essential to modulate H_2_O_2_-induced cell necrosis and activation of AMPK is involved in the process.

## 2. Materials and Methods

### 2.1. Astrocyte Culture

Astrocytes were isolated from early postnatal cortices (P1–P3). All the brain from littermates of heterozygous mating was processed. The details were described previously [9]. Briefly, cortices were dissected and triturated in cold HBSS. Cell suspension was diluted to fresh HBSS, treated with trypsin-EDTA and passed through a 40 *μ*m cell strainer. Cells were spun down and resuspended in prewarm DMEM media (GIBCO) supplemented with 10% FBS and seeded 2 × 10^6^ cells into flasks. The medium was replaced every two days. Cell cultures were maintained prior to experiments. Confluence of the astrocyte culture was usually 70%–90% before the experiments were performed. And one should make sure all the experiments were carried out on confluent astrocytes. All the chemicals were purchased from Sigma Aldrich unless indicated otherwise.

### 2.2. Cytotoxicity Assay

The cytotoxicity of H_2_O_2_ was measured by MTT assay as previously reported [10]. Briefly, 1 × 10^4^ astrocyte cells were seeded onto 96-well microplates and one kept growing until confluent stage. The cells were washed briefly and resuspended in medium with indicated concentrations of H_2_O_2_ for 45 min. 20 *μ*L of 5 mg/mL MTT in PBS working solution was added to each well and incubated at 37°C for another 4 hours. The optical density at 570 nm was measured with microplate reader. Cell viability was calculated as a percentage of viable cells in H_2_O_2_-treated groups versus untreated control.

### 2.3. Cx43 Gap Junction Blocker, AMPK Inhibitor, and Transient siRNA Transfection Treatment

In order to measure the effect of gap junction blockers on H_2_O_2_-induced cell death, gap junction Cx43 blocker carbenoxolone (Cbx) at the concentration of 100 *μ*M was added 30 min earlier before H_2_O_2_ treatment. AMPK inhibitor, Compound C (Comp C), at the concentration of 10 *μ*M was added 30 min earlier as well. Transient siRNA transfection was used to knock down Cx43 as described protocol [11].

### 2.4. Measurement of Reactive Oxygen Species (ROS) Production

ROS level was measured using 2,7-dichlorofluorescein diacetate (DCFDA) fluorescence dye as described [12]. Cells were treated with 1 *μ*M DCFDA at 37°C for 30 min and passed through a 40 *μ*m cell strainer. Fluorescence intensity was measured by flow cytometry (FACS Calibar; Becton-Dickinson). The numbers of each analysis containing at least 1 × 10^4^ cells were quantified and analyzed using CellQuest software according to the manufacture.

### 2.5. Western Blotting Analysis

Astrocytes were digested with 0.25% Trypsin/EDTA and resuspended in RIPA Lysis Buffer (50 Mm Tris-HCl pH 8.0, 150 mM NaCl, 1% Nonidet P-40, 0.5% sodium deoxycholate, 0.1% sodium dodecyl sulphate (SDS), 1 mM sodium orthovanadate, and protease inhibitors tablet from Roche). The samples were put on ice for 30 min and then centrifuged at 14,000*g* for 25 min at 4°C. The supernatants were subjected to protein concentration assay by Bradford methods [13]. Equal amount of 100 *μ*g protein was loaded to SDS-PAGE gel and transferred to nitrocellulose membrane, subject to a standard protocol by blocking and antibody treatment [14]. Briefly, the nitrocellulose membrane was blocked in 5% nonfat milk for 1 hour, subject to primary antibody overnight, and the membrane was washed three times in PBS. Antibodies including Cx43 (1 : 1000), GAPDH (1 : 1000) (Neomarkers), and p-AMPK and AMPK (Santa Cruz) were used here. A horseradish peroxidase-conjugated second antibody was added and the membrane was incubated 1 h, followed by ECL chemoluminescence system. The intensity of the signals was semiquantified by ImageJ software, downloaded from the National Institute of Health (USA) (NIH Image-free download available at https://imagej.nih.gov/ij/download.html). The relative expression was normalized to GAPDH or AMPK expression [15].

### 2.6. Cell Death and Apoptosis Analysis

DAPI and TUNEL double staining were used here for cell death analysis. The result was visualized under an inverted fluorescence microscope. Random fields of view photographed were selected. Cell death was determined and expressed as percentage TUNEL positive cells of total cells, which was determined by DAPI staining.

The apoptosis and necrosis analysis has been done previously by flow cytometry method by Annexin V propidium iodide (PI) detection [16]. The cells were washed with ice-cold PBS and resuspended in binding buffer at the concentration of 1 × 10^6^ cells/mL. Staining was done in dark for 15 min and then immediately analyzed using flow cytometry (FACS Calibur; Becton-Dickinson). 1 × 10^4^ cells of each analysis were quantified and analyzed using CellQuest software according to the manufacturer.

### 2.7. Statistical

The results were presented as the average ± standard deviation (SD). Student's *t*-test was used to evaluate the response to a change in conditions. *P* < 0.05 is considered significant (*∗*); *P* < 0.01 is considered highly significant (*∗∗*).

## 3. Results

### 3.1. H_2_O_2_ Decreased Cell Viability and Expression of Cx43, Inducing Cell Death in Astrocytes

**Figure 1:**
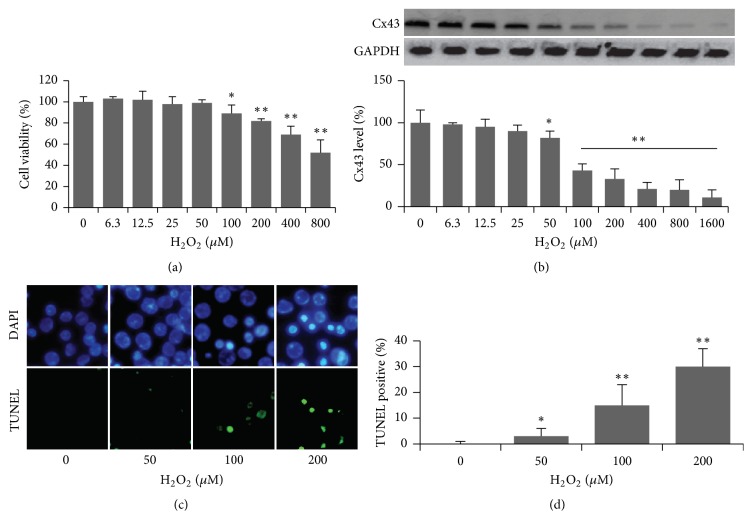
H_2_O_2_ decreased cell viability and expression of Cx43, inducing cell death in astrocytes. (a) Cytotoxicity of H_2_O_2_ in MTT assay. (b) Presentative bands and quantification expression of Cx43 by western blotting. (c) Cell death was measured by DAPI and TUNEL costaining. Scale bar: 10 *μ*m. (d) TUNEL labeling cells were quantified by counting cells from random fields (*n* > 300 cells). *∗* indicated *p* < 0.05; *∗∗* indicated *p* < 0.01.

To determine the cytotoxic effects of H_2_O_2_ in astrocytes, MTT assay was used. Cells were exposed to various concentrations of H_2_O_2_ as shown in Figure 1(a). Cytotoxic effect is weak if the concentration of 100 *μ*M H_2_O_2_ was treated. Thus, the concentration more than 100 *μ*M was highly significant compared with control group.

Astrocytes were treated with H_2_O_2_ for 45 min in serum-free medium and then harvested immediately after treatment, while untreated (control) cells were maintained in the same condition. There was a twofold reduction in Cx43 level with 100 *μ*M H_2_O_2_ treatment, and the expression decreased with increasing H_2_O_2_ treatment, and the level was further quantified in the histogram shown in Figures 1(b) and 1(c).

The result demonstrated that cell had a marked resistance to low concentration of H_2_O_2_. Exposure of cells to 200 *μ*M H_2_O_2_ resulted in morphological changes as well as cell damage, including DNA fragmentation and nuclear condensation, Figure 1(d). TUNEL positive cells were further quantified in Figure 1(d). Taken together, these results suggested H_2_O_2_-induced astrocytes injury and death was specific, in a concentration dependent manner, and it is likely to involve in Cx43 in the mediation.

### 3.2. siRNA Knockdown or Blocking Cx43 by Cbx Enhanced H_2_O_2_-Induced Necrosis in Astrocytes

**Figure 2:**
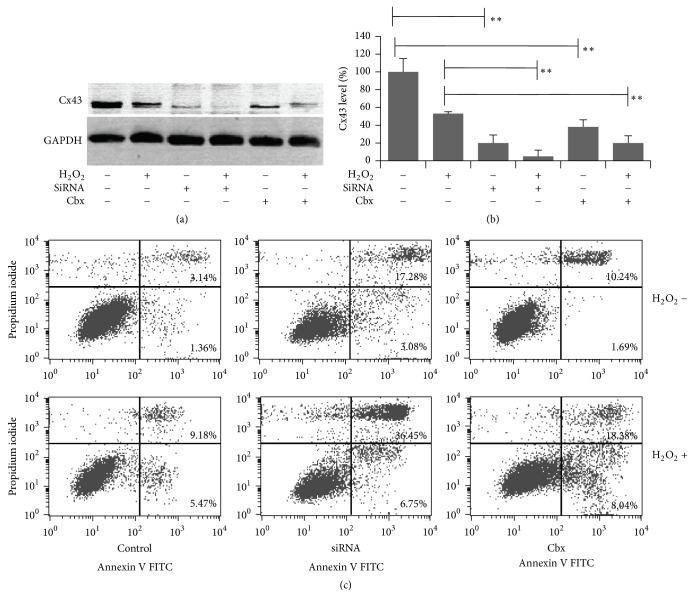
siRNA knockdown or blocking Cx43 by Cbx enhanced H_2_O_2_-induced necrosis in astrocytes. (a) Presentative bands expression of Cx43 was detected by western blotting. (b) Quantification of Cx43/GAPDH by membrane intensity. (c) Necrosis and apoptosis cells were stained by Annexin V FITC and PI and determined by flow cytometry (*n* = 10,000 cells). *∗* indicated *p* < 0.05; *∗∗* indicated *p* < 0.01.

It has been previously reported that Cx43 leads to a protection effect in apoptosis and necrosis in primary astrocytes [17]. However, H_2_O_2_-induced cell damage in astrocytes has not been well quantified. To determine whether functional Cx43 expression is important for protection against H_2_O_2_, astrocytes were exposed to the Cx43 channel blockers Cbx or transfected with siRNA to knock down Cx43. The results confirmed that siRNA knockdown or its inhibitor Cbx decreased Cx43 expression in astrocytes. H_2_O_2_ treatment further enhanced this effect of Cx43 reduction, Figures 2(a) and 2(b). Secondly, it is not clear whether the decreased Cx43 expression in astrocytes is essential for their resistance to H_2_O_2_-induced apoptosis and necrosis. The flow cytometry method for double staining was used here. siRNA knockdown or Cbx treated in astrocytes produced more necrosis cells, 17.28% and 10.24% of the total number of cells, respectively. While exposed to 100 *μ*M H_2_O_2_, both necrosis and apoptosis cells were produced. Astrocytes lacking Cx43 exhibited 35.45% and 18.38% cell necrosis at this concentration. Only 9.18% of astrocytes exposed with H_2_O_2_ were necrosis as shown in Figure 2(c). The enhanced percentage of necrosis is correlated with Cx43 reduction. This result indicates that Cx43 deficient astrocytes are more sensitive to H_2_O_2_-induced necrosis. Taken together, it suggests that expression of Cx43 is important for cell survival, especially to necrosis in response to H_2_O_2_.

### 3.3. siRNA Knockdown or Blocking Cx43 by Cbx Enhanced H_2_O_2_-Induced ROS Production

**Figure 3:**
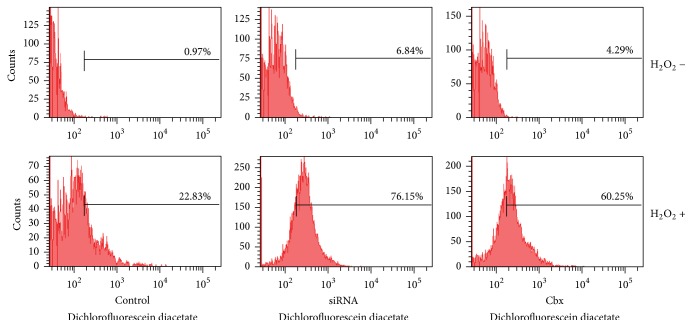
siRNA knockdown or blocking Cx43 by Cbx enhanced H_2_O_2_ induced ROS production in astrocytes. ROS generation (%) was measured using ROS-sensitive fluorometric probe 2,7-dichlorofluorescein diacetate (DCFDA) by flow cytometry.

H_2_O_2_ is well known to produce free radicals to inhibit gap junctional intercellular communication [18]. Next, how Cx43 expression contributes to the H_2_O_2_-induced ROS was determined. As shown in Figure 3, astrocytes lacking Cx43 exhibited 6.84% and 4.29% ROS production at this concentration, while it increased to 76.15% and 60.25% combined with H_2_O_2_ treatment, which is significantly higher than the 22.83% in the control group. Here, the data indicated that H_2_O_2_ increased ROS production, and reduction of Cx43 enhanced it.

### 3.4. Attenuating H_2_O_2_-Induced Necrosis in Astrocytes Was Involved in AMPK Signal Pathway

**Figure 4:**
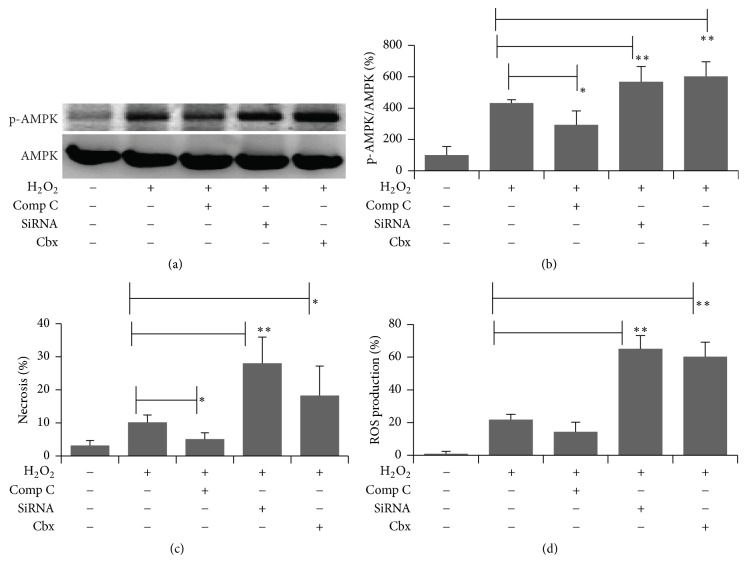
Attenuating H_2_O_2_-induced necrosis in astrocytes cell was involved in AMPK signal pathway. (a) H_2_O_2_-induced p-AMPK signal protein expression. (b) Quantification of p-AMPK/AMPK by membrane intensity. (c) Necrosis was determined using Annexin V FITC and PI double staining by flow cytometry. (d) ROS production was measured using DCFDA staining by flow cytometry. *∗* indicated *p* < 0.05; *∗∗* indicated *p* < 0.01.

At present, the signaling pathway that is involved in ROS production has been investigated, and AMPK is an important sensor that controls various signaling pathway [19]. Here, the effect of H_2_O_2_ on AMPK activation was examined. As shown in Figures 4(a) and 4(b), H_2_O_2_ treatment leads to an increase of AMPK phosphorylation, but AMPK inhibitor Comp C blocks this phosphorylation. Note that reduction of Cx43 activated the phosphorylation even further. To address whether AMPK activation correlated with H_2_O_2_-induced necrosis and ROS production, flow cytometry was used and the data was quantified in Figures 4(c) and 4(d). Interestingly, Comp C treatment also markedly inhibited H_2_O_2_-induced necrosis and ROS production. Taken together, these data suggest that AMPK is involved in H_2_O_2_-induced cell necrosis and ROS production.

## 4. Discussion

It is well known that H_2_O_2_ exposure increases ROS production inside cells. In this paper, astrocytes under oxidative challenge with H_2_O_2_ resulted in decreased cell viability, which is consistent with previous studies in control vehicle-treated cultures [20]. Cell damage, including DNA fragmentation and nuclear condensation, was also observed [20].

Here, the relationship between H_2_O_2_ exposure and Cx43 was investigated. Cx43, one of the predominant connexins for astrocytes, decreased expression at a dose-dependent manner. In addition, the TUNEL straining result indicated the apoptosis phenotype. These observations indicate that H_2_O_2_-induced injury was specific and might be correlated with Cx43 expression level.

To this end, siRNA target Cx43 gene or pharmacologically blocking Cx43 expression was tested. It is not surprising to see the decreased Cx43 expression. However, the question here is that H_2_O_2_-induced cell injury was not clear. The flow cytometry analysis with double staining could well distinguish cell apoptosis and necrosis. The results indicated that H_2_O_2_ induced both cell apoptosis and necrosis. Multiple cascade kinases are involved in those processes [21], while knockdown or blocking Cx43 resulted in mainly cell necrosis, as well as a small portion apoptosis. Thus, Cx43 protected against H_2_O_2_-induced necrosis, indicating that antinecrosis effect of Cx43 expression was present in normal astrocytes.

Increased Cx43 expression as a potential mediator to improve the neuroprotective activity was also widely investigated. The ROS production was another important index to the homeostasis. Environment stress leading to ROS increased dramatically, which resulted in significant damage to cells. Here, ROS production was increased, and inhibition of Cx43 expression enhanced the production. This result is similar to cell apoptosis and necrosis assay, which suggested the crucial role of Cx43 in both processes. Effect of ROS on cell metabolism including apoptosis is well documented in a variety of species [22].

**Figure 5:**
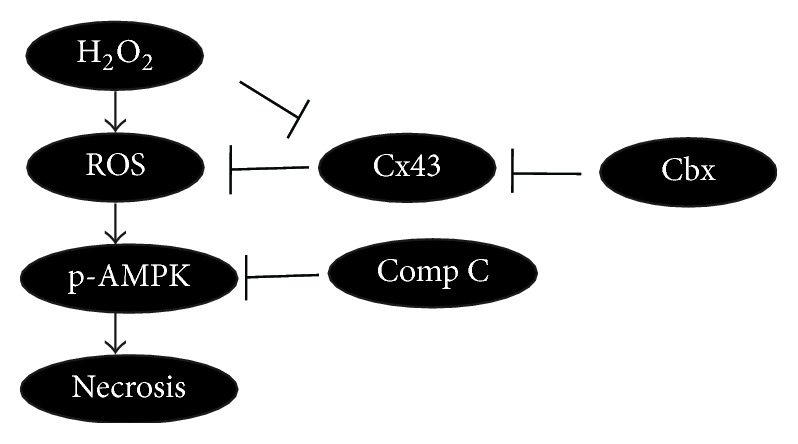
Molecular mechanism model. Cx43 plays its role in H_2_O_2_-induced ROS production and necrosis through activation of AMPK in astrocytes.

Although H_2_O_2_ affected cell necrosis and ROS production significantly, less was known about its relation with AMPK that plays a leading role in cellular energy homeostasis. Astrocytes treated with H_2_O_2_ activate AMPK phosphorylation, which results in increasing of cell necrosis as concluded in Figure 5. We observed that AMPK activation after H_2_O_2_ treatment could be inhibited by its inhibitor Comp C, which significantly reduced cell necrosis and ROS production by H_2_O_2_. It suggests that AMPK activation favors cell death in ROS stressed. Similarly, we also found that knockdown Cx43 by siRNA or by its inhibitor Cbx could significantly enhance H_2_O_2_-induced damage. These data strongly argued that Cx43 had shown resistant effect in H_2_O_2_ stressed astrocytes and it is AMPK phosphorylation dependent.

In all, our study adds a novel connection to the mechanism of AMPK phosphorylation and cell apoptosis and necrosis in Cx43-regulated H_2_O_2_ resistance. We show that Cx43 expression plays a crucial role in protection against H_2_O_2_ injury. Several of the key points have been reported previously, which include H_2_O_2_-treated decreased cell viability, the reduction expression of Cx43 expression, and the neuroprotective actions of Cx43. Our data link these previous observations into a new view. Further experiments are needed to well elucidate the important function of Cx43 as an intracellular signaling molecule, with the differential sensitivity of Cx43 overexpressing astrocytes and downstream gene under AMPK triggered in this process.
